# How hospital top managers reason about the central leadership task of balancing quality of patient care, economy and professionals’ engagement: an interview study

**DOI:** 10.1108/LHS-02-2022-0009

**Published:** 2022-12-27

**Authors:** Fredrik Bååthe, Mia von Knorring, Karin Isaksson-Rø

**Affiliations:** Institute for Studies of the Medical Profession, LEFO, Oslo, Norway; Institute of Stress Medicine at Region Västra Götaland, Gothenburg, Sweden and Institute of Health and Care Sciences, Sahlgrenska Academy at Gothenburg University, Gothenburg, Sweden; Department of Learning, Informatics, Management and Ethics, Medical Management Centre, Karolinska Institutet, Stockholm, Sweden; Institute for Studies of the Medical Profession, LEFO, Oslo, Norway and Department of Behavioural Sciences in Medicine, Institute of Basic Medical Sciences, Medical Faculty, University of Oslo, Oslo, Norway

**Keywords:** Health leadership competencies, Leadership, Management development, Working conditions, Qualitative research, Quality of working life

## Abstract

**Purpose:**

This study aims to deepen the understanding of how top managers reason about handling the relationships between quality of patient care, economy and professionals’ engagement.

**Design/methodology/approach:**

Qualitative design. Individual in-depth interviews with all members of the executive management team at an emergency hospital in Norway were analysed using reflexive thematic method.

**Findings:**

The top managers had the intention to balance between quality of patient care, economy and professionals’ engagement. This became increasingly difficult in times of high internal or external pressures. Then top management acted as if economy was the most important focus.

**Practical implications:**

For health-care top managers to lead the pursuit towards increased sustainability in health care, there is a need to balance between quality of patient care, economy and professionals’ engagement. This study shows that this balancing act is not an anomaly top-managers can eradicate. Instead, they need to recognize, accept and deliberately act with that in mind, which can create virtuous development spirals where managers and health-professional communicate and collaborate, benefitting quality of patient care, economy and professionals’ engagement. However, this study builds on a limited number of participants. More research is needed.

**Originality/value:**

Sustainable health care needs to balance quality of patient care and economy while at the same time ensure professionals’ engagement. Even though this is a central leadership task for managers at all levels, there is limited knowledge about how top managers reason about this.

## Introduction

To create a more sustainable health care, there is a need to address the quality of patient care and economy while at the same time ensure professional well-being and engagement for the health-care workforce (Bodenheimer and Sinsky, 2014; Baathe *et al.*, 2019; Rø *et al.*, 2020; Swensen and Shanafelt, 2017; Løvseth and de Lange, 2020; Linzer *et al.*, 2017; Storkholm *et al.*, 2017). Managers have the formal role and responsibility to handle these relationships, and this is a central leadership task at all levels (Andersson, 2015; Shanafelt *et al.*, 2015; Baathe and Norback, 2013; Shanafelt *et al.*, 2021).

Although these relationships have been discussed and are well known, there seems to be an overarching need, and at the same time, an inherent difficulty, in handling all aspects at the same time. A review from 2013 found that 70% of the interventions aiming to improve quality and reduce health-care costs did not succeed in doing both (Hussey *et al.*, 2013). Common strategies, such as hospital or department mergers and downsizing, failed to attain increased quality of care (Leatt *et al.*, 1997) and instead led to negative effects on work environments, as well as increased stress, burnout and feelings of alienation among employees (Bourbonnais *et al.*, 2005; McKinlay and Marceau, 2011; Nordang *et al.*, 2010).

Research has found clear associations between professionals’ engagement and multiple measures of care quality (including patient experiences of care, doctors experience of care and quality of care linked to outcomes) (Firth-Cozens and Greenhalgh, 1997; West *et al.*, 2009; Shanafelt *et al.*, 2002; Angerer and Weigl, 2015). Studies show how an increased focus on production volumes and economy has a negative impact on professionals’ engagement (Le Grand, 2003; Eijkenaar *et al.*, 2013; Lindgren *et al.*, 2013; Khullar *et al.*, 2015; von Knorring *et al.*, 2016).

Leading hospitals is a highly complex task (Glouberman and Mintzberg, 2001a; Plsek and Wilson, 2001). Research on the manager role in health care has identified the need for new approaches (Porter and Lee, 2013; Snell *et al.*, 2011; Dickson and Owen, 2016; Reinertsen *et al.*, 2008; Greenhalgh and Papoutsi, 2018; Kuhlmann and von Knorring, 2014; Glouberman and Mintzberg, 2001b; Porter, 2010), with the common denominator “to fix healthcare”, as expressed by Mintzberg (2011). While managers most likely aspire to handle the inherent complexity arising from the relationships between the aspects, it seems from the prevailing health delivery research that something is amiss. In 2017, the National Academy of Medicine launched a national burnout crises in the USA (NATIONAL ACADEMY OF MEDICINE, 2017). In 2019, the UK created a similar initiative to tackle clinical burnout among health-care professionals (The King´s fund, 2019). In Norway, there is reduced professional satisfaction among doctors since 2010 (Rosta *et al.*, 2020).

Some researchers describe this negative development spiral in healthcare as a “vicious pattern” (Swensen and Shanafelt, 2020; Storkholm *et al.*, 2017; Rø *et al.*, 2020; Shanafelt, 2021).

Research about finding compromises, balancing desired objectives against one another when all cannot be met, suggest managers need to invest time to ensure they and the health professionals talk more *with* each other (and less *about* each other) (Mowles, 2021; Stacey, 2011; Schein, 1999; Schein, 2017; Baathe *et al.*, 2019). This will create a sense of partnership that can contribute with changes meeting multiple demands. Organizational leaders need to deliberately collaborate closer with the health-care professionals to create practical and balanced solutions (Shanafelt, 2021). Storkholm *et al.* (2017) provides a clinical example were seemingly incompatible objectives where fruitfully handled in collaboration between the middle managers and the clinicians. Management was able to create clinical engagement for quality improvement by translating the managerial task of reducing the budget into a change process that resonated with the professionals’ mindsets. In this collaborative change process, professional engagement, quality of care and economy were all addressed. Swensen and Shanafelt show several practical examples of how hospital middle managers systematically can involve the employees and work together to balance potentially conflicting goals in organizational development (Swensen and Shanafelt, 2020). They demonstrate how collaboration between managers and clinicians to improve the workplace, and related clinical care is a way to balance dynamic tensions and ensure professionals’ engagement. These types of deliberate collaborations leading to fruitful results can be called “virtuous patterns” (Storkholm *et al.*, 2017; Swensen and Shanafelt, 2020; Rø *et al.*, 2020; Kegan and Lahey, 2016).

There is thus growing research about how middle managers’ different strategies and actual ways-of-working when handling these relations is paving the way towards a more sustainable health care ( Løvseth and de Lange, 2020; Dickson and Owen, 2016; Swensen and Shanafelt, 2020; von Knorring, 2012; Storkholm *et al.*, 2019; Baathe and Norback, 2013). Although handling the relationships between quality of patient care, economy and professionals’ engagement is a central leadership task for managers at all levels, and there is limited knowledge about how top managers reason about this (Dyrbye *et al.*, 2017; Sinsky *et al.*, 2013). This study is contributing with knowledge to fill that research gap.

The aim of this study is to deepen the understanding of how top managers reason about handling the relationships between quality of patient care, economy and professionals’ engagement.

## Material and methods

We chose a qualitative study design since there is a need to deepen the knowledge in this area of inquiry (Patton, 2002; Greenhalgh *et al.*, 2004; Malterud, 2014).

### Participants and sampling

In-depth interviews were performed between March and June 2018 with the five top managers forming the executive management team at one mid-sized emergency hospital in Norway. The interviewed participants included all the members from the hospitals’ top management team. The participants occupied the following positions: chief executive officer, chief medical officer, human resources director, finance director, quality director, information technology (IT) director and chief of staff. Two of the participants held two positions. Two were women and three were men. Three of the interviewees had a clinical background, one as a physician and two as nurses. The other two had an educational background in business administration, with extensive work experience in health care.

### Data collection

To provide a trustful setting and facilitate open information sharing, the interviews were conducted at the hospital in the local office of each top manager. The interviews lasted between one and two hours. We followed a semi-structured interview guide to enable consistency while allowing the interviewers to probe aspects arising spontaneously during the interview (Kvale and Brinkmann, 2009). Each interview started with a question about what the current position is and what it entails. Then the respondents were asked to describe a day when they felt satisfied or really fulfilled at work, followed by a question to describe a day when they experienced work was hard. After this, they were asked to reflect on the relationships between quality of patient care, economy and professionals’ engagement. All interviews were digitally recorded and transcribed verbatim.

### Data analysis

A reflexive thematic analysis approach was used to analyse the data (Braun and Clarke, 2006; Braun and Clarke, 2020). In this approach, new knowledge is the end result of an iterative inductive process where the researchers’ experience is seen as an analytical resource, as well as their reflexive engagement with data and interpretation. This involved paying close attention to the empirical interview material and striving towards identifying and making sense of patterns of meaning across the empirical material (Braun and Clarke, 2020).

In the first step of the analytical procedure, data familiarization, the verbatim transcripts of the interviews were read, and the managerial statements relating to the study aim were tagged with preliminary descriptive codes. This was done with the aim of guiding the readings and focusing on what was being discussed in the interview material in preparation for the systematic data coding (Braun and Clarke, 2020). The second step generated initial themes from coded and collated data. These initial themes were compared with each other to further develop more abstract categories, by reviewing and combining themes. These themes were, in turn, further refined and defined. In this step of the analytical process, themes were being named and the most informative citations were being selected to each theme. When naming the final themes, an inclusive description to convey the multifaceted meaning was aimed for.

During the analytical process, the transdisciplinary group of authors worked in parallel to enrich the empirical interpretations and reduce the risk of any one author overpowering the empirical voices (Patton, 2014). The interview material was first to read individually, and individual interpretations were presented to the other researchers. Different understandings or additional nuances were compared and contrasted, leading to a substantiated understanding of identified patterns across the empirical material. Alternative interpretations were continuously sought in critical reflections.

The tentative themes and preliminary results were presented to a larger group of researchers in the same research area during an international conference. The interpretations made sense and were considered useful to others, and insights from this exchange were integrated when writing up the result. This study has an interpretative qualitative research approach (Patton, 2002).

In the results section, information-rich quotations are used to illustrate the findings. Each interview was given an identification number, and the number within the parentheses indicates the interview from which the quotation was taken. The symbols […] and [] indicate, respectively, omissions and additions to the transcript excerpts. Changes have been made solely to protect anonymity or enable readability without altering the meaning of the content. All quotations presented are translated from Norwegian.

During the writing process, the results have been presented to the interviewees, who recognized the findings and found them meaningful. This connects to what Miles and colleagues consider a sign of quality in qualitative research (Miles *et al.*, 2013).

## Findings

This study explored how top managers reason about handling the relationships between quality of patient care, economy and professionals’ engagement.

The interviewed top managers stressed their intention of handling these dimensions in relation to each other, while at the same time pointing to the difficulties with upholding this intention.

In the following, we first present how the top managers reasoned about what helped them to uphold their intention. Then we present candid accounts about problems arising when working towards this intention.

### Managerial reasoning about handling the relationships

#### Creating a shared understanding of how each person can contribute to hospital goals.

Creating a shared understanding around the hospital goals was considered central for the interviewed top managers when striving towards handling the tensions arising from multiple demands. To encourage employees to become more involved in meeting the overarching goals of the hospital and to have people from different departments working together around this was a central perspective of top management. It would drive increased cooperation, which was thought to improve quality. One manager expressed it this way:

Just to get our employees more involved in our thoughts and our visions and in the business, I think that would contribute to having a better and closer cooperation between groups. And if one could get a better and closer cooperation across groups, I also think we would be able to increase the quality of care. (IP2)

The top managers also mentioned the importance of establishing a shared understanding with their own superiors, the hospital board. The interviewees expressed the need to experience that the hospital board recognized and supported how they, as top managers, carried out their work – for example, that the board acknowledged the importance of them collaborating with the employees to bring about engagement for the changes necessary to achieve both the short- and the long-term goals of the hospital. One top manager described it this way:

It is not evident that pushing forward with short-term measures [which the board advocates] will have significant long-term effects. We have to make sure that we have the organisation with us, and that we make the right moves. (IP 4)

#### Acknowledging the imbalances and recognizing we are in this together and there will always be challenges.

One way the top managers reasoned related to acknowledging and conveying to the employees how good patient quality is linked to good financial quality and how catering to both aspects simultaneously when engaging in development work contributes to professional well-being and engagement. This is how one top manager put it:

We have mentioned many times that when there are change processes, improvement work, quality development work – you always need to consider the economical perspective. […] Which elements contribute both to better patient care and better professional fulfilment? When you find flow and manage to create an effective treatment course for individual patients that is also effective from a resource’s perspective, with no major bottlenecks – well, when you have that experience of flow, you also contribute to a healthy economy. That is so obvious! (IP 4)

A top manager talked about how managers must dare to address the discrepancy between different expectations while at the same time acknowledge that we are all in this together. It was considered important to address that there always will be challenges since the demands for certain treatment can be much higher than the economic possibilities, and this imbalance negatively impacts the relation between employees and managers:

But the expectations are so much higher, and the demands are so much higher than the economic resources in relation to the present situation. We need to dare to address this discrepancy and how it impacts us and how we can approach it. (IP5)

To involve all employees in handling the hospital challenges, the importance of experiencing a manageable work situation, with basic structures in place, i.e. the tasks that need to be performed and how to adapt when someone calls in sick, was part of top managers reasoning. One top manager said this had been in place for nurses and other health professionals for a long time but was still lacking for doctors:

I think we need good planning, also for doctors. What tasks do we need to cover and how do we actually do it, and what do we do when someone is sick or at home with sick children. […] If we do not take care of that, I think that affects doctors’ job satisfaction, along with the quality of service and patient care. (IP 3)

### Managerial reasoning hindering the intention of balancing

Despite the top managers intentions and efforts to work actively to handle the relationships between quality of patient care, economy and professionals´ engagement, it was clear that they also struggled with this.

#### Difficulties in handling multiple perspectives when experiencing increased pressure.

Holding multiple perspectives in the mind simultaneously was presented as challenging. It was not uncommon to totally have to change perspectives during the day. One top manager exemplified this with one meeting having focus on planning for expanding the hospital capacity to meet a growing population in the area, and then the next meeting was about cutting down on the number of employees due to budget restrictions:

But of course – there we sit and work with views and visions about increased population area for the hospital […] expansions in surgery, medicine, in geriatric psychiatry, mental health care etc. And directly afterwards you go into the next meeting where you will sit down and discuss how you can cut staff numbers, how we can streamline care and how we can reduce number of beds so that we can manage to meet budgets. (IP2)

A manager described how different mindsets created tension when talking about the need to improve on the economy. The hospital board requested fast actions to handle the numbers. At the same time, the top managers strove towards collaborative change processes focusing quality of care and allowing ample time for the care professionals to be engaged in the process:

I have a board that I answer to. The board has stressed that we are in a crisis, and I need to show them that things are changing […] I will do so, but for me, it´s more important to reach the goal in two and a half years than to do so in three months. (IP 5)

A manager described the difficulties with balancing the often-extensive external investigations with the need to be focused on the different demands arising from the internal operations:

Leading a modern hospital means that outside of the internal operational tasks, there are also a number of external tasks being asked for from official bodies […] Our regional health trust and the national health department are experts in expecting large demanding investigations to be completed in record time. These are often unrealistic or inhuman demands that are linked to certain tasks. (IP 2)

A top manager provided a recent example from a larger development project when he eventually resorted to positional power to accelerate the pace of change. He stressed the difficulties of handling professional involvement (satisfaction), quality of care and resources (including time and economy) in ongoing development work when pressure (internal and/or external) increased.

I really tried to work in a visionary way, focusing on the goal, and brought in a unit manager who had much legitimacy in the organisation to lead that work. […] We had a lot of staff working groups discussing how we ourselves should take hold of the future and make the necessary changes. And then too many people came up with arguments for why it would not work, why it was impossible. So, I decided that I would decide on some changes, and then we will work on how to implement the change, instead of talking about whether we should make the change or not. I said that the zero alternative, i.e., to continue as before, is no longer an alternative. (IP 5)

#### Expecting clinical employees to understand the connection between good clinical care and good economy.

One manager pointed to the paradoxical challenge with hospital management, where it can feel out of place to talk about economy, when the overall focus in a hospital needs to be the quality of patient care:

That is what is difficult, I find, with running a hospital, because it´s medicine […] our basic tasks in hospital. It´s out of place to talk about money, or resources. (IP1)

Another manager described the striving to motivate employees, and how focus on economy is difficult to use in motivational work when the mindset of the health-care professionals is not about economy but providing good care to the patients:

Motivational work is demanding, because […] nobody works here because of hospital economy. And then I tried, because I really wanted not to talk about, not to say the word economy at all […] but this time just focus on the other dimensions and all the good things we do (IP5).

The top managers described several situations in which they observed that the clinical department managers did not fully recognize how economic issues needed to be considered in all development work. Top managers described how they sometimes needed to move economy to centre stage, with quality of patient care and professionals’ satisfaction becoming subordinate to budgetary concerns. One top manager reasoned as follows:

What’s terrible about economy is that if you do not include those perspectives in the development work, then suddenly the priorities change so that finances come first before both physician satisfaction and patient treatment. So, then development is, in a way, driven by economy. (IP 4)

The top managers described how they, despite the intention to collaborate about clinical changes, found they sometimes did not succeed in conveying a reason to change that resonated enough with the clinical employees to create engagement and drive towards the change. Employees expressed worries about possible reduced quality after the change process, while top managers saw the risk of having to close down activities completely if change processes were not instituted. One interviewee expressed it like this:

We need to stop thinking risk and rather talk about uncertainty. And we must dare to talk about that uncertainty […] And it's very difficult. Because rapidly someone will say, “no, I don’t dare to do that”. And then it is either the value argument or the patient safety trump card that is brought forward. And, if patient safety is to be used as an argument to not explore another way of working, I will bring forward the hospital’s financial perspective. Well, what would it be like if we were forced to close [a certain part of the hospital]? (IP 5)

One manager talked about a collaborative development process where the employees and the clinical department managers were involved, and where it, in the end, became difficult to be the top manager with a responsibility to hold up several perspectives:

[…] we agreed; “ok, we take as our starting point some of the clinical things. What do we need to develop, what can we improve?” Then we came to the end of the session and then we kind of said “yes - and how is the economy, how do we ensure that the economy is in this?”. And suddenly […] it was like “no way! […] are we going to start talking economy now? Now that we were finally getting somewhere? No, damn it, we are not!” (IP4)

A top manager talked about the difficulties in communicating the important task of balancing the different aspects. The manager explained how they indirectly mean clinical care when talking about economy. They, as top leaders, have made a choice to try to talk less about the budgetary numbers, at the same time, the yearly employee survey, year after year, indicates that the employees consider top managers mostly talk about economy:

When we speak about economy, we actually indirectly mean clinical care. But it doesn´t seem to be perceived that way in the employee surveys from year to year. I see that they [the employees] answer in that way, although I know that we have talked not only about money. But they perceive it that way and why do they? Because they do not want to pay too much attention to economy. They think we should pay attention only to clinical issues, because that ought to be the hospital focus. (IP 1)

An additional example from the interviews of how budgetary issues tended to come to the forefront for top management concerned the focus in the individual and regular monitoring meetings with the clinical department managers. In these meetings, the top managers expressed that they used most of the time focusing on budget numbers. They seemed to take quality for granted and think that clinical quality would always be a priority for the clinical department managers and, therefore, needed less attention in the monitoring meetings:

In our monitoring conversations [with clinical department managers], we expect everyone to know about the quality goals, but we focus and talk mostly about economic issues. We are more concerned with these. (IP 1)

## Discussion

This study explored how top managers reason about handling the relationships between quality of patient care, economy and professionals’ engagement to learn more about hinders and facilitators towards creating more sustainable healthcare.

When the top managers reasoned about handling quality of patient care, economy and professionals’ engagement, they stressed their intention of handling the aspects in relation to each other. At the same time, they pointed to the difficulties with this, especially with communication about economy, and how they resorted to use positional powers to speed up the change and how the intention of handling the aspects in relation to each other then was replaced with a single focus on economy.

That top managers centred their reasonings about the importance of balancing quality of patient care, economy and professionals´ engagement was not a surprising finding as it corresponds well with modern leadership theory and concepts from management literature (Kouzes and Posner, 2006; Reinertsen, 2008; Snell *et al.*, 2011; Mintzberg, 2011). The top managers in this study accentuated the importance of creating a shared understanding of how each person contributes to the hospital goals and the necessity for management and employees to find common ground, and engage in the balancing together, when meeting successive challenges.

Kegan and Lahey provide support to the importance of the interviewed top managers’ intention to balance between quality of patient care, economy and professionals’ engagement (Kegan and Lahey, 2016). They suggest that business excellence and employee engagement need not be at odds but can be combined in such a way that each causes the other to flourish. They suggest that an employee experience of meaningfulness through engagement in change processes is vital – also for the economy (Kegan and Lahey, 2016). This is echoed by Isaksson-Rø *et al.*, who suggest engaging the health professionals in improving the clinical care processes to better meet the needs of patients, is the only long-term sustainable way to handle budgetary dilemmas in health care (Rø *et al.*, 2020).

A more surprising and interesting finding was how the top managers described how it can feel paradoxical to talk about economy when the hospital goal should be quality of care and how they realized that the clinical staff were not motivated by economy. At the same time, they pointed to the importance of talking about and thinking about economy, and how they expected the clinical staff to understand that when they, as top managers, talked about economy, they actually meant quality of care.

There is limited support for the assumption that clinicians intuitively will understand that talking about and focusing economy actually implies a focus on quality of care. Research about finding compromises and balancing seemingly contradicting objectives, stresses the importance of talking with each other to find solutions that meet multiple demands (Stacey, 2011; Mowles, 2021). At the same time, this research is clear about how the outcome from any change initiative cannot be known in advance and how this experience of uncertainty of outcome can be highly taxing (ibid). Mintzberg and Glouberman (2001a, 2001b) discuss the fact that the expression of medical mastery within the professional group of physicians can be interpreted as if they also are skillful in their way of relating to organizational changes. Research suggests that is not the case. On the contrary, literature suggests that physicians sometimes show organizational illiteracy (Simpson, 1975; Baathe and Norback, 2013). The findings point to the need for top managers to work thoughtfully with the next level of managers, potentially using the well-established principle of teaching in the medical community, see one, do one, teach one. Now with the intention of teaching health-care managers how to thoughtfully integrate economy in the clinical development work.

Another interesting finding was how the top managers described the difficulties with holding on to the intention of employee involvement, when experiencing increased internal or external pressure. They explained how the combination of parallel multiple processes (planning for increased capacity and number of beds while at the same time reducing number of employees), the board’s impatience regarding economic cut-backs, and the health trusts expectations to completing huge extra tasks in short time, made it difficult to keep up a collaborative communication to ensure employee involvement and reaching a shared understanding between health professionals and management. They candidly described how they sometimes resorted to using positional power to try to increase the speed of change, retreated from trying to balance the multiple aspects and instead had a single focus on economy.

The literature has described the difficulties with communication since there often is a lack of shared goals between the different actors in health-care settings. Glouberman and Mintzberg (2001a) divide health-care actors into four categories; care (nurses), cure (doctors), control (management) and community (society), and describe how perspectives and goals for health care are different between these actors and how this almost creates different “languages”. It is considered to facilitate communicating within each category but communicating across categories is considered difficult. In the same vein, Skirbekk *et al.*, described how the clinician perspective often concerns giving the individual patient the best possible treatment, while managers often are concerned about giving the population the best possible treatment, by increasing patient flow and keeping budgets balanced (Skirbekk *et al.*, 2017). Knowledge about these well-known communicational difficulties can be important for top managers when working towards having collaborative change processes. Storkholm *et al.*, refer to a clinical change process, at a clinic in Denmark, where managers in healthcare explicitly, in their communication with clinical employees, first addressed how change initiatives would improve clinical care (Storkholm *et al.*, 2017). When that was clearly established among the employees, engagement towards the change followed and the manager could help guide the improvement process such that it also contributes to improved economy. Via this active employee involvement in the change process, both improvement in the quality of care, professionals’ engagement and budgetary goals were reached (Storkholm *et al.*, 2017). Research suggests that being clear about how changes contribute towards better patient care quality is one key way for leaders in healthcare to ignite a more virtuous development spiral (Swensen and Shanafelt, 2020; Rø *et al.*, 2020). Top managers need to actively use this knowledge and initiate fruitful collaborations that can contribute towards realizing the intention of balancing quality of patient care, economy and professionals’ engagement.

## Conclusions

Handling quality of patient care and economy while at the same time ensuring professionals’ engagement is a central leadership task. The findings show that this handling is an act of balancing that requires continuous attention by top management. It is not a stable relationship that is either achieved or not. While top managers stress the importance of balancing, it seems increasingly difficult when coming under pressure. During such times, they tend to retreat towards a focus on economy. Top managers need to be explicit about the connections between patient care quality and economy, in their collaboration and communication, to ensure professionals’ engagement.

However, this study builds on a limited number of participants, and more research is needed.

## Practical implications

For health-care top managers to lead the pursuit towards increased sustainability in health care, there is a need to balance between quality of patient care, economy and professionals’ engagement. This study shows that this balancing act is not an anomaly top-managers can eradicate. Instead, they need to recognize, accept and deliberately act with that in mind, which can create virtuous development spirals where managers and health-professional communicate and collaborate, benefitting quality of patient care, economy and professionals’ engagement.

## Limitations and future research

This study has a major limitation in that the empirical material was only based on interviews with five top hospital managers. As such, we suggest the findings from this study to be taken as indicative and that more research is needed. At the same time, it is worth noticing that the five participant includes all members of the hospital’s executive management team and as such, the empirical material reflect the full top managers complexity. The empirical material is rich, and we suggest this study contributes with meaningful knowledge despite the limited number of interviewees. This is in line with Malterud *et al.*, who argue that also few information-rich interviews can contribute with new, meaningful knowledge (Malterud *et al.*, 2016).

In this study, we examined top managers’ perceptions about their work situation, without observing their actual behaviour. Therefore, a weakness concerns whether the interviewed top managers described their actual reality. We could not be sure about that; however, previous research has found that what people present in interviews generally reflects their perceptions, and these perceptions also inform their actions (Czarniawska, 2004).

Consistent with other qualitative research, the findings from this study can be useful to other healthcare delivery organizations experiencing similar challenges in their specific contexts, helping them to make better sense of their own situations (Larsson, 2009; Malterud, 2014; Patton, 2014). However, this study builds on a limited number of participants and more studies on top managers are needed.
